# Ten Lifestyle Modification Approaches to Treat Atrial Fibrillation

**DOI:** 10.7759/cureus.2682

**Published:** 2018-05-24

**Authors:** Syed Rafay Ali Sabzwari, Lohit Garg, Dhanunjaya Lakkireddy, John Day

**Affiliations:** 1 Cardiology Fellowship, Lehigh valley health network, Allentown, USA; 2 Cardiology, Lehigh valley health network, Allentown, USA; 3 Electrophysiology, Kansas University Medical Center, Kansas City, KS.; 4 Electrophysiology, Intermountain Medical Center

**Keywords:** atrial fibrillation, lifestyle modification

## Abstract

Atrial fibrillation (AF) is the most common arrhythmia affecting three million people in the United States (US). Across different races in the US, the incidence of other races was comparable to that of Caucasian population. This points towards the importance of certain lifestyle modifications that can help prevent and treat this disorder. This article discusses 10 such factors. Smoking has been linked to AF, with almost 36% risk reduction if quit. Hypertension has 56% increased risk of atrial fibrillation in which the role of lifestyle changes is well known. Similarly, alcohol-induced atrial fibrillation has 10% increased risk of atrial fibrillation. On the other hand, several case reports document red bull as the cause of atrial fibrillation. Moreover, the risk of atrial fibrillation is four times higher in patients with obstructive sleep apnea (OSA) independent of other confounding variables. Additionally, it has been shown that acute sleep deprivation increases AF risk by 3.36 times. Furthermore, diabetes mellitus and obesity also contribute greatly to atrial fibrillation. This risk has been shown to be around 40% more with diabetes. Diet itself has an impact: numerous studies have shown Mediterranean diet to reduce the risk of AF and cerebrovascular accident in addition to olive oil, fruits and vegetables. Even emotions are important with 85% less AF on ‘happy days’. Needless to mention, yoga has been well demonstrated to have almost 24% reduction in AF. Similarly, physical activity in all forms is beneficial. In summary, lifestyle modifications reduce the incidence of AF, induce more AF remission and also produce successful ablation outcomes.

## Introduction and background

Atrial fibrillation (AF) is the most common arrhythmia affecting an estimated three million people in the United States (US) and 20 million people around the world [1]. When there is increasing prevalence of atrial fibrillation across the world, it is interesting to note that the epicenter of this disease is North America. Given such a high disease burden in North America, it is important to analyze the contributing factors that separate out North America from the rest of the world. This article is an analysis of such factors.

Chugh et al. compared the incidence between Asia and North America and reported up to a 10 times higher incidence of AF in North America (19.8 vs 196.3 per 100,000 person-years for women and 33.8 vs 264.5 per 100,000 person-years for men) [1]. However, in another study when 1.4 million medicare patients having 375,000 incident episodes of AF over 3.2 years were studied with respect to different races, the incidence (per thousand person-years) of other races was comparable to that of the Caucasian population; Caucasian (35.7), African American (28.2), Hispanic (26.9), Asian (26) [2]. Hence, these results argue that genetic factors alone cannot explain the underlying cause for prevalence variation in North America compared to the rest of the world.

The question then is whether this is due to lifestyle differences or a reporting bias. While arguments can be made in both directions, it is realistic to understand that lifestyle factors do play an important role. The reporting bias can be excluded to some degree by the fact that some countries, like Japan, have a very robust reporting system for atrial fibrillation. Recently, there have been many studies evaluating lifestyle modification and atrial fibrillation. The key studies will be discussed in this article.

## Review

Smoking

Smoking has clearly been linked to atrial fibrillation. In one study, current smokers had a 2.1 times increased risk and former smokers a 1.3 times increased risk of AF. Fortunately, in a study of 15,792 patients, those who were able to quit smoking decreased their AF risk by 36% [3]. The mechanisms involved likely include the inflammatory role of nicotine, relationship of smoking to coronary artery disease and chronic obstructive pulmonary disease (COPD).

Hypertension

Hypertension is another factor that is well known to contribute to atrial fibrillation with one large study reporting a 56% increased risk [4]. Another study finds hypertension to be the most common elevated risk factor for AF in their participants with the incidence of 38% at age 55 years, 57% at 65 years and 70% at 75 years [5]. The manifestation of atrial fibrillation could be considered part of the spectrum of end-organ damage due to hypertension. Hence controlling hypertension may indirectly reduce chances of developing AF.

Alcohol

**Figure 1:**
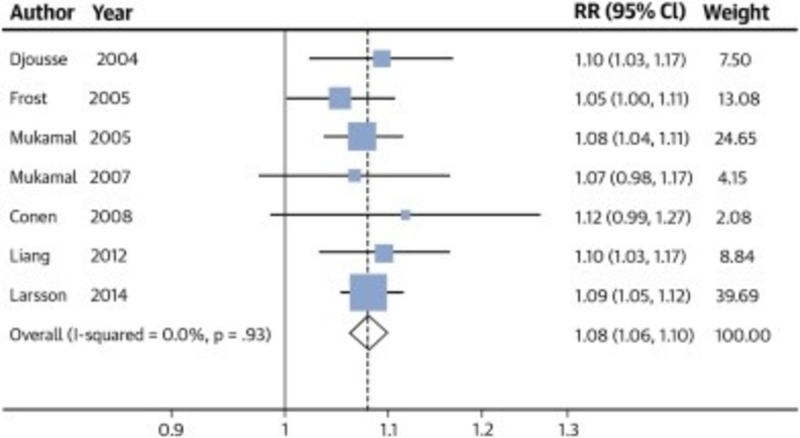
Comparison of studies on the effect of once a day alcohol on risk of developing atrial fibrillation.

It is common to find patients presenting to the emergency department with atrial fibrillation after excessive consumption of alcohol. This, ‘holiday heart’ phenomenon has been well documented in the medical literature. Staerk et al. found lifetime risk for AF to be 40.9% (95% CI: 36.1-45.7) for those with increased alcohol consumption compared to 35.1% (95% CI: 32.2-38.0) for those with no alcohol consumption at age 55 years [5]. In a recent large meta-analysis, even one daily drink in five out of eight studies showed a statistically significant increased risk of atrial fibrillation. Overall, this amounts to approximately a 10% increased risk of atrial fibrillation (Figure 1) [6].

Stimulants

Stimulants are an important AF trigger. There have already been several case reports that document Red Bull as the cause of atrial fibrillation [7]. To explore this association further, there is even a trial currently underway. It is interesting to differentiate stimulants based on natural or artificial sources. For example, the majority of studies now show no link between natural sources of caffeine, such as coffee, tea and chocolate, and AF. In contrast, artificial sources of caffeine, as well as stimulants found in over-the-counter and prescription medications, may be AF triggers.

Sleep deprivation

Sleep deprivation, whether from obstructive sleep apnea (OSA) or just not sleeping long enough, may be another modifiable AF risk factor. The risk of atrial fibrillation is four times higher in patients with OSA independent of obesity, age, hypertension, heart failure or other confounding variables. In addition, 49% patients of AF have OSA [8].

Many studies have evaluated the link between OSA and AF. For example, in one study of 400 patients with moderate to severe OSA, 24-hour Holter monitoring showed AF in 3% of patients which is three times higher than the general prevalence [9]. Preceding hypopneas, before paroxysms of AF shown in a case cross design, supports an immediate causal temporal relationship.

Even more significant is the increasing data implicating OSA as a risk factor for recurrence of AF after ablation or cardioversion. In a prospective study of 720 patients by Neilan et al., the presence of OSA (hazard ratio (HR): 2.79, CI: 1.97-3.94, p < 0.0001) and untreated sleep apnea (HR: 1.61, CI: 1.35-1.92, p < 0.001) were highly associated with AF recurrence [10]. The same study showed a reduction in blood pressure, atrial size and ventricular mass in patients treated for OSA. Similarly, a meta-analysis showed that in patients undergoing pulmonary vein isolation or medical management for AF, there was a 42% relative risk reduction in AF recurrence with the use of continuous positive airway pressure (CPAP) [11, 12]. This means that AF treatment outcomes may be ineffective if OSA is not adequately treated. Thus, OSA should not just be considered a causative factor for AF but a continued insult in the natural progression of AF.

Even if patients do not suffer from OSA, not sleeping long enough may contribute to AF. For example, one study showed that acute sleep deprivation increases AF risk by 3.36 times. As reports show that Americans may be sleeping up to two hours less per night than what was common in the 1960s, this may be yet another potential modifiable risk factor in the AF epidemic [13].

Diabetes mellitus

**Figure 2:**
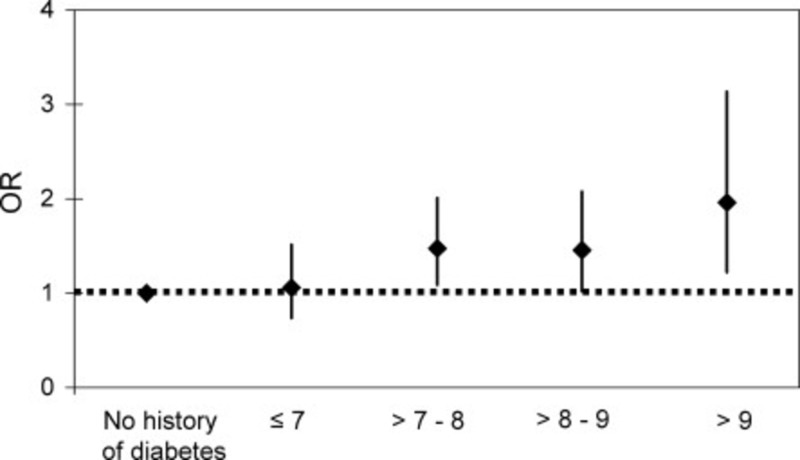
Relationship between A1c and risk of developing atrial fibrillation.

**Figure 3:**
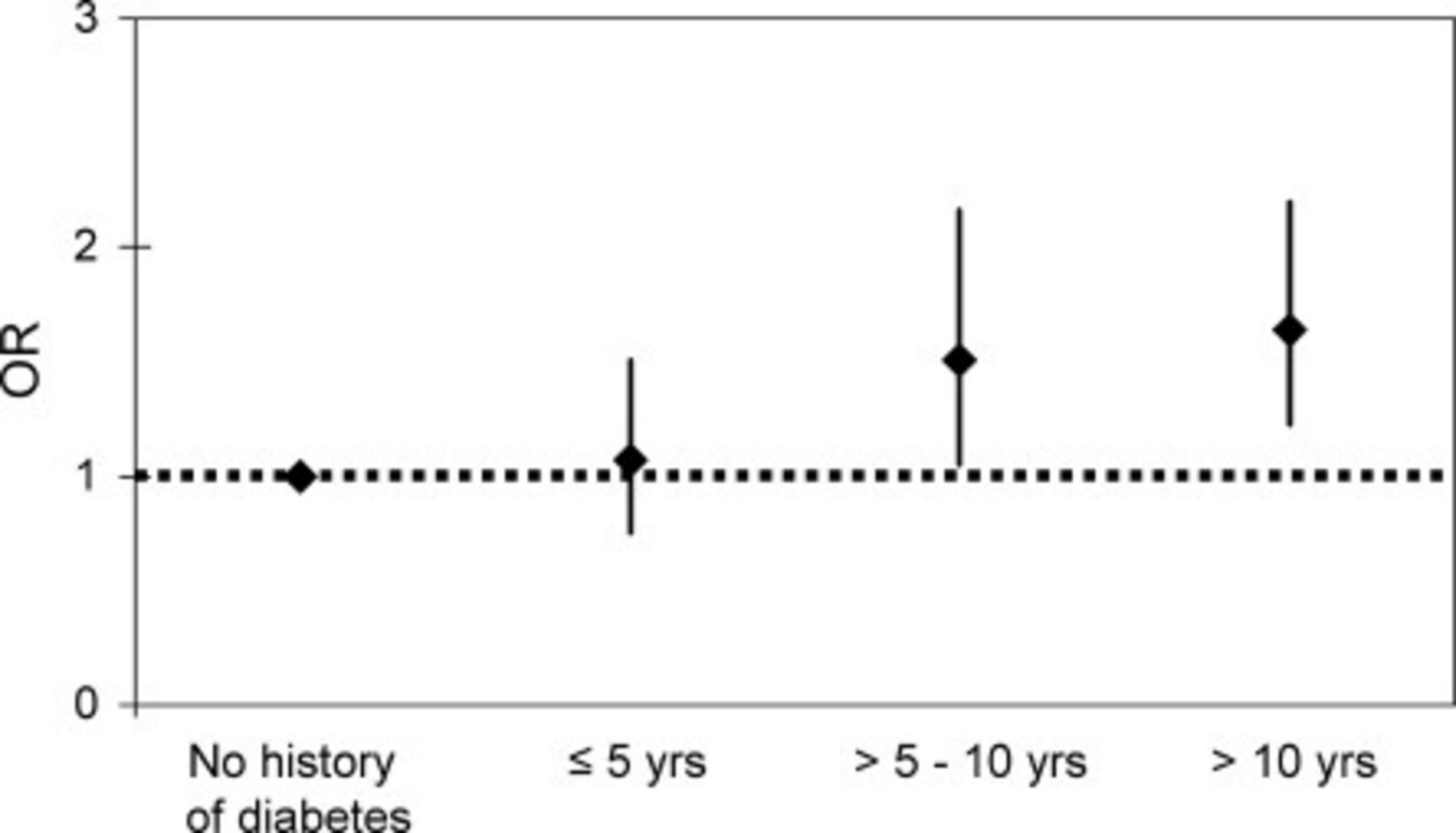
Relationship between duration of diabetes mellitus and risk of developing atrial fibrillation.

The multi-system adverse effects of diabetes mellitus may also contribute to an increased risk of atrial fibrillation. This risk has been shown to be increased by approximately 40% with diabetes. As with AF, there has also been a diabetes epidemic with diabetes rates increasing by 75% over the last 20 years [14]. Interestingly, one study showed that the higher the hemoglobin A1c (Figure 2) and the longer the duration of diabetes (Figure 3), the higher the risk of atrial fibrillation [14]. Diabetes also has a direct and indirect correlation with other AF comorbidities that include obesity and diet.

Diet

**Figure 4:**
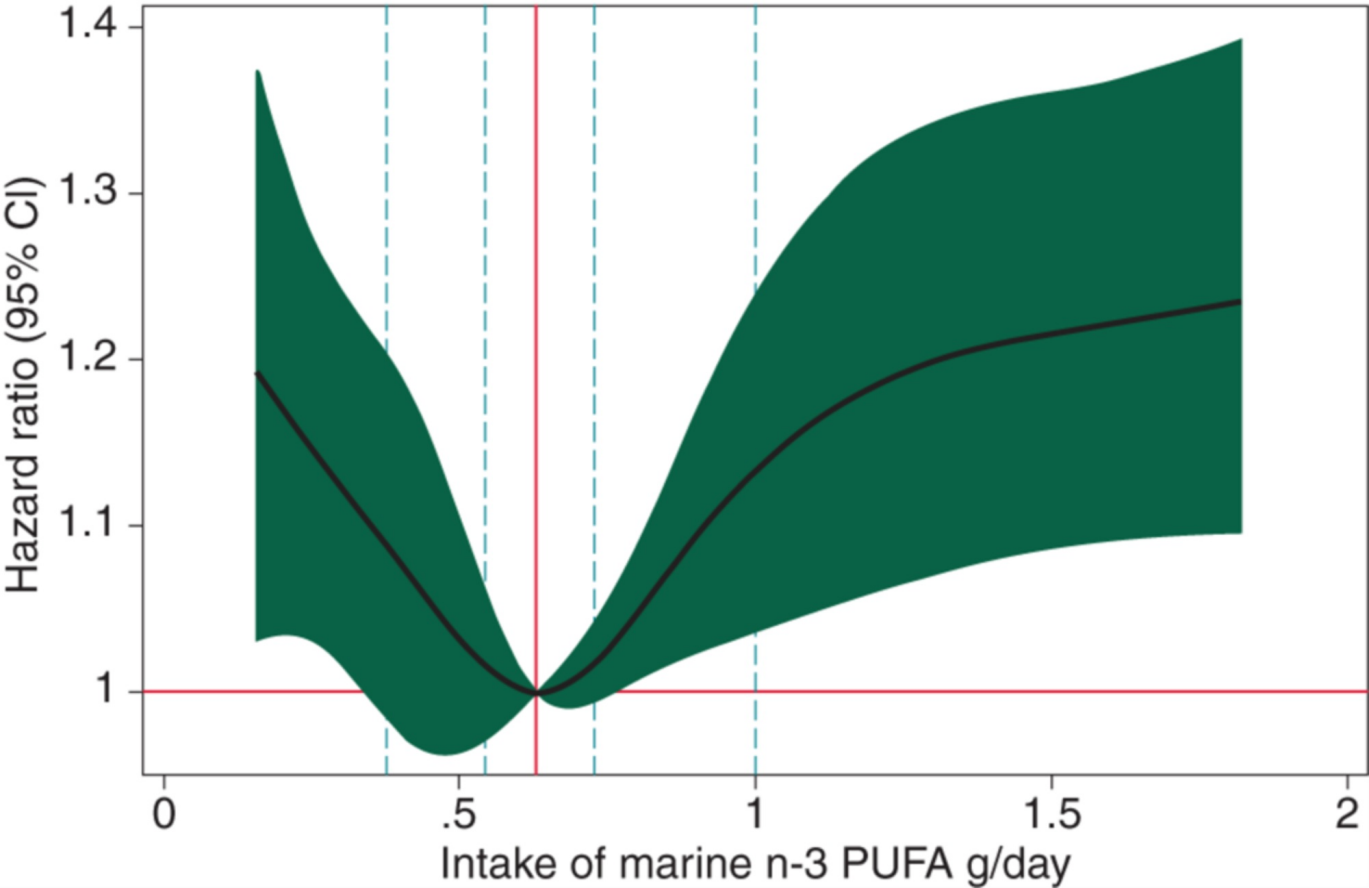
A U-shaped association between consumption of n-3 fatty acids and development of atrial fibrillation.

Diet is a critically important component of lifestyle modification. Numerous studies have shown that the Mediterranean diet may reduce the risk of AF and cerebrovascular accident [15]. The traditional Mediterranean diet is primarily plant-based with fish and no significant amount of added sugars or processed foods. In addition, olive oil, nuts, high fruit and vegetable consumption also decreases AF due to the presence of natural antioxidants [16, 17]. Interestingly, the fish component of the Mediterranean diet containing a significant amount of n-3 fatty acids shows a ‘U’ shaped curve with too little or too much of diet showing negative correlation with AF (Figure 4) [18].

Obesity

A natural consequence of an unhealthy diet is obesity. Not surprisingly, the US has both the highest burden of obesity and AF. At the cornerstone of weight loss to treat AF is the 'Long-Term Effect of Goal-Directed Weight Management in an Atrial Fibrillation Cohort: A Long-Term Follow-Up Study' (LEGACY) [19].

The LEGACY Study included a total of 355 AF overweight patients with an average body mass index (BMI) of 27. While they were waiting for their AF ablation procedure, 38% were successful in losing approximately 36 pounds. Even this modest weight loss showed a phenomenal AF reduction. Indeed, nearly half the patients went into complete remission, with no further need of antiarrhythmics or ablation, from weight loss alone. In addition, the systolic blood pressure decreased by 18 mmHg, the C-reactive protein (CRP) dropped by 76%, diabetes went into remission in 88% of patients, the low-density lipoprotein (LDL) dropped by 16% and triglycerides by 31%, echocardiographic abnormalities resolved, and there was a 200% improvement in the sense of well being [19].

Healthy mindset and stress management

Alongside a healthy diet is the importance of a healthy mindset in treating AF. From a well-known study comes evidence of 85% less AF on ‘happy days’ [20]. In descending order, sadness, anger, stress, impatience and anxiety have shown association. Interestingly, hunger was not shown to increase AF risk in this study.

Needless to mention, yoga has been well demonstrated to have an almost 24% reduction in AF. In this study, the most beneficial effect of yoga was in the reduction of symptomatic AF episodes (3.8 pre-yoga vs 2.9 post-yoga) followed by symptomatic non-AF episodes (2.1 vs 1.4) with only a slight reduction in asymptomatic AF episodes (0.12 vs 0.04) [21]. The mechanisms affiliated with yoga include stress management, better emotional well being, and reduction in inflammation.

Physical activity

**Figure 5:**
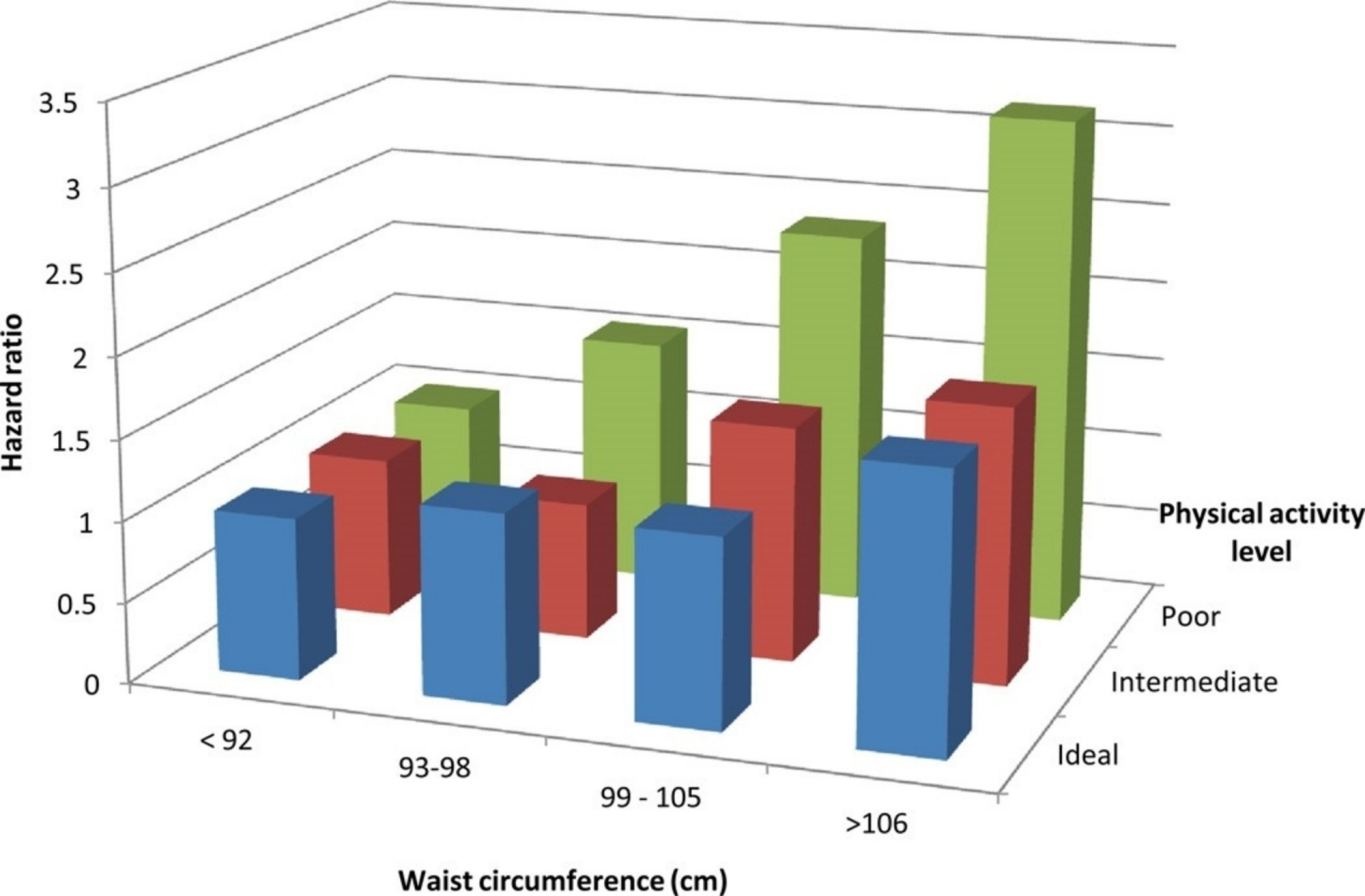
Relationship between physical activity, waist circumference and risk of developing atrial fibrillation.

Furthermore, not just yoga but physical activity in any form reduces the risk of atrial fibrillation. It has been shown that the higher the waist circumference the more impact physical activity has in reducing the hazard ratio shown in a study of 14,219 patients shown in Figure 5 [22, 23].

From getting up and walking around to formal exercise daily, physical activity in all forms is beneficial. The only exception is for ultra-athletes involved in strenuous competitive exercises. For example, in a Swedish study comparing extreme exercise and AF amongst Swedish cross-country skiers enrolling 52,755 participants, there was a 30% increased AF risk with faster times and more races [24]. Similar results have been seen from other ultra-endurance events.

## Conclusions

In summary, AF is the most common arrhythmia, particularly in the developed world. For countries like the US, lifestyle factors trump genetics. The fact that patients can prevent or modify the progression of AF has profound implications on reducing disease burden.

The lifestyle interventions discussed in this article, even if adopted in moderation, can help significantly. For those patients who are unable to completely reverse AF with lifestyle modification, these lifestyle changes will likely enhance AF treatment efficacy. Therefore, both internists and cardiologists need to become more aware and proactive in initiating early adoption of lifestyle modification to prevent and treat AF.
